# Anti-Metastatic Benefits Produced by Hyperthermia and a CCL3 Derivative

**DOI:** 10.3390/cancers11111770

**Published:** 2019-11-11

**Authors:** Liqiu Ma, Ryosuke Kambe, Tomoko Tsuchiya, Shiro Kanegasaki, Akihisa Takahashi

**Affiliations:** 1 Gunma University Heavy Ion Medical Center, Gunma 371-8511, Japan; maliqiu@ciae.ac.cn (L.M.); m1600505@gunma-u.ac.jp (R.K.); 2 China Institute of Atomic Energy, Beijing 102413, China; 3 Research Institute, National Center for Global Health and Medicine, Tokyo 162-8655, Japan; ttsuchiy-tky@umin.ac.jp (T.T.); profkane@ims.u-tokyo.ac.jp (S.K.)

**Keywords:** hyperthermia, malignant tumor, metastases, eMIP, chemokine variant, high-frequency waves, anti-metastasis

## Abstract

Significant numbers of malignant tumor cells that have spread to surrounding tissues and other distant organs are often too small to be picked up in a diagnostic test, and prevention of even such small metastases should improve patient outcomes. Using a mouse model, we show in this article that intravenous administration of a human CCL3 variant carrying a single amino acid substitution after mild local hyperthermia not only induces tumor growth inhibition at the treated site but also inhibits metastasis. Colon26 adenocarcinoma cells (1 × 10^5^ cells/mouse) were grafted subcutaneously into the right hind leg of syngeneic BALB/c mice and after nine days, when tumor size reached ~11 mm in diameter, the local tumor mass was exposed to high-frequency waves, by which intratumoral temperature was maintained at 42 °C for 30 min. Mice received the CCL3 variant named eMIP (2 μg/mouse/day) intravenously for five consecutive days starting one day after heat treatment. We found that tumor growth in eMIP recipients after hyperthermia was inhibited markedly but no effect was seen in animals treated with either hyperthermia or eMIP alone. Furthermore, the number of lung metastases evaluated at 18 days after hyperthermia treatment was dramatically reduced in animals receiving the combination therapy compared with all other controls. These results encourage future clinical application of this combination therapy.

## 1. Introduction

Hyperthermia (HT) has been used as a cancer treatment for decades to enhance the efficacy of low-dose radiotherapy or chemotherapy [1,2,3,4]. HT has been shown to inhibit cellular invasion into the extracellular matrix or surrounding tissues, at least in some cancer types [5]. However, control of “distant” metastases from aggressive cancers by any means remains an important therapeutic challenge [6]. 

Immunotherapy is known to work synergistically with other therapies to improve efficacy [7,8,9], and HT combined with immunotherapy has been shown to enhance anti-tumor activity [10,11,12,13]. HT itself can modulate the immune system [11] by enhancing dendritic cell activity and inducing anti-tumor-specific cytotoxic T cells [10]. Thus, it may be possible to control metastasis by augmenting the immune system after local HT treatment at the primary tumor site. 

Antitumor radiation efficacy and the abscopal effect are enhanced by a recombinant variant of CCL3 [13], now called eMIP. HMGB1 and HSP70 released in the tumor bed from overexpressed tumor cells following irradiation were reported to trap injected eMIP and work together with eMIP in complexes [14]. These complexes played a key role in recruiting and activating tumor inhibitory NK cells and CD4^+^ and CD8^+^ T cells [13,14]. Since mild heat shock (42 °C) was shown to enhance HSP70 significantly in colon carcinoma in vivo [15], we anticipated that a combination treatment of HT with eMIP may produce strong anti-tumor activity.

In this study, we show that eMIP administration after mild local HT treatment not only induces tumor growth inhibition at the treated site but also produces strong metastasis inhibition.

## 2. Results

### 2.1. eMIP Administration after Mild Local HT Treatment Induces Anti-Tumor Activity at the Treated Site

**Figure 1:**
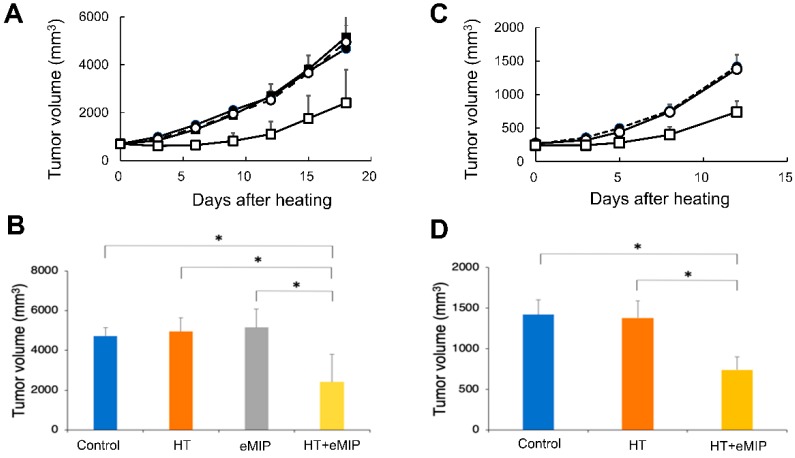
Effects of hyperthermia (HT), the CCL3 variant named eMIP and their combination on tumor growth. (**A**) Time course of tumor growth. Colon26 adenocarcinoma cells were implanted subcutaneously in the right hind leg of BALB/c mice. After nine days when tumor size reached ~11 mm in diameter, the tumor mass was heated using a radio-frequency device. Intratumoral temperature was maintained at 42 °C for 30 min (day 0). eMIP (2 μg/mouse) was administered intravenously once a day for five consecutive days beginning on the day following heat treatment. Symbols: Control (●), eMIP (■), HT (○), HT + eMIP (□). (**B**) Tumor volume at day 18 post heat treatment. Mean values (*n* = 6) with standard error are shown. * *p* < 0.05 (ANOVA). (**C**) Time course of tumor growth. Colon26 cells were injected subcutaneously in the right flank. After 16 days, the tumor mass (~11 mm in diameter) was heated using a heating device made in-house. Skin surface temperature was maintained at 42 °C for 30 min (day 0). eMIP (2.5 μg/mouse) was administered intravenously once a day for five consecutive days starting from the day following heat treatment. Symbols: Control (●), HT (○), HT + eMIP (□). (D) Tumor volume at day 12 post heat treatment. Mean values (*n* = 7) with standard error are shown. * *p* < 0.05 (ANOVA).

We examined the effect of combination treatment with HT and eMIP on tumor growth. Colon26 adenocarcinoma cells subcutaneously implanted in the right hind leg of BALB/c mice were allowed to grow for nine days. The tumor mass (~11 mm in diameter) was exposed to high-frequency waves using an 8-MHz capacitive, radio-frequency device. Intratumoral temperature was maintained at 42 °C for 30 min. From the next day, eMIP (2 μg/mouse) was intravenously administered once a day for five consecutive days. Under these conditions, no detectable difference in tumor growth was found between control (no treatment) and heat treatment groups (Figure 1A). However, administration of eMIP to HT-treated animals induced marked tumor growth inhibition (Figure 1A,B), while eMIP alone did not influence tumor growth.

A similar observation was made when Colon26 cells were implanted subcutaneously in the right flank and treated with HT and eMIP under similar conditions, except in this case an electromagnetic heating device made in-house was used to maintain skin surface temperature of a subcutaneously growing tumor at 42 °C for 30 min. The skin surface temperature of the tumor mass was monitored using a disposable surface temperature probe. As shown in Figure 1C,D, HT + eMIP markedly inhibited tumor growth, whereas no difference was found between HT and control (no treatment) groups. It is important to note that in this case, intratumoral temperature was likely to be significantly lower than 42 °C as the skin surface was heated, and that even then, tumor growth was inhibited by eMIP combined with HT. 

### 2.2. eMIP Administration after HT Treatment Inhibited Lung Metastasis

**Figure 2:**
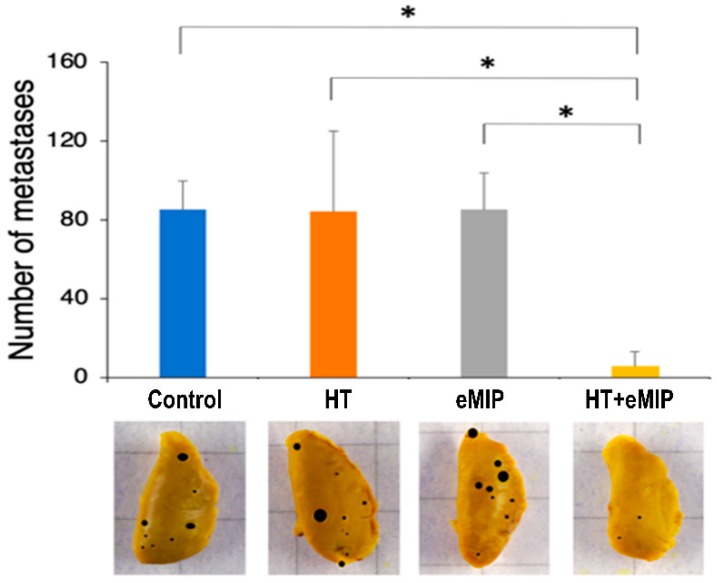
Numbers of lung nodules at 18 days after HT treatment. (**Top**) Mean numbers of metastatic nodules. Error bars show standard deviation (*n* = 6). * *p* < 0.05 (ANOVA). (**Bottom**) Photographs of left upper lobe from control, HT, eMIP and HT + eMIP group mice are shown as representatives.

At the end of the experiment shown in Figure 1A,B, we evaluated the number of lung metastases by counting metastatic nodules macroscopically on the surface of all pulmonary lobes. As shown in Figure 2, more than 80 metastatic nodules were found in control (no treatment), and in HT and eMIP only groups at 18 days after HT treatment. In contrast, mice in the HT + eMIP group had significantly fewer nodules. Total number of nodules was six at most in three mice, and none in the remaining three mice of the six tested.

## 3. Discussion 

Since invasion into adjacent tissues and distant metastasis are known to be the cause of 90% of cancer-related deaths [16], a feasible and effective method to prevent metastasis is required to improve patient outcome. In the present study, we showed using a model animal that a combination of HT + eMIP dramatically reduced the incidence of lung metastases. In half of the animals tested no metastasis was found in any lung lobe. Beside metastasis inhibition, tumor growth at the heat-treated site was inhibited markedly by the administration of eMIP under conditions where HT treatment or eMIP itself did not influence tumor growth. It was shown by Adkins et al. that mild heat shock (42 °C) did not inhibit proliferation of murine colon cancer cells [15], while Kanegasaki et al. showed that eMIP itself did not influence tumor growth [14]. To our knowledge, this is the first report that shows a chemokine playing a significant role in induction of anti-tumor and anti-metastatic effects in combination with HT treatment.

**Figure 3:**
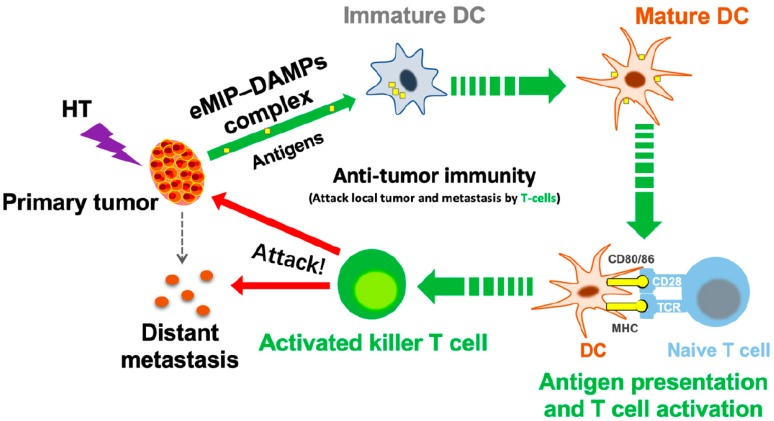
A possible mechanism of action of the present combination therapy. DAMPs, such as HSP70 and HMGB1 are released from HT-treated tumor cells, which form a complex with intravenously administered eMIP. The complex activates dendritic cells followed by T cell activation. Finally, activated killer T cells attack tumors at the treated site and at distant metastases.

In similar studies using a 6 MeV electron beam instead of HT, eMIP was shown to be effective in inhibiting various syngeneic tumors including MethA fibrosarcoma (BALB/c) and Lewis lung carcinoma (C57BL/6), in addition to Colon26-derived tumors [14]. Damage-associated molecular patterns (DAMPs) or alarmins such as HSP70 and HMGB1 released by tumor cells following irradiation work synergistically with eMIP to recruit and activate killer T cells [13,14]. Since HSP70 is known to be produced by mild heat-shock (42 °C)-treated CT26 colon cancer cells [15], a similar common mechanism might work following combination of HT and eMIP (Figure 3). If so, such a combination treatment could be applicable to treat any kind of malignant tumor, whether carcinoma or sarcoma.

Clinically, radiofrequency hyperthermia (RHT) has widely been used as adjuvant therapy combined with chemotherapy or radiotherapy [17,18]. Until now, however, most animal studies used a water bath as a heat source due to difficulty in obtaining proper heating instruments for use in small animals [19,20,21,22,23]. Even using water-bath HT, the target temperature inside the tumor is lower than the skin outside of the tumor margin [24]. In the present study, however, we used a heating device made in-house, or a newly developed RHT device suitable for animal studies, by which temperature in the surrounding normal tissues can be kept lower compared with the tumor area. We believe that temperature control in the present study is more accurate and effective, and that surrounding tissues were protected from scalding.

## 4. Materials and Methods

### 4.1. Preparation of Chemokine

**Figure 4:**
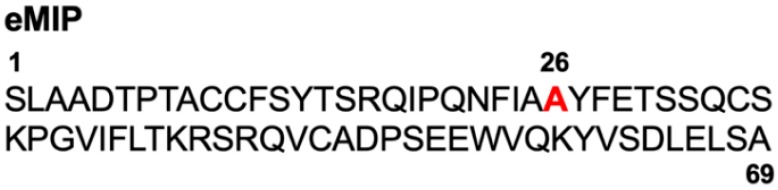
Amino acid sequence of eMIP.

Recombinant eMIP, a 69-amino-acid variant of human CCL3 carrying a single amino acid substitution (red in Figure 4) was generated and purified as described previously [13]. This variant has a reduced tendency to aggregate compared with parental CCL3.

### 4.2. Mice

Six-week old female (Figure 1A,B) or male BALB/c mice (Figure 1C,D) were purchased from Nippon SLC (Hamamatsu-shi, Shizuoka, Japan) and housed in a barrier system with controlled light (12L:12D) and temperature (22 ± 2 °C). They were fed a diet of mouse chow and water ad libitum, and used at 7-weeks old. All animal experiments were carried out in accordance with the guidelines for animal experiments at our university (No. 17-036).

### 4.3. Tumor Cells 

Colon26 adenocarcinoma cells were provided by the Cell Resource Center for the Biomedical Research, Institute of Development, Aging and Cancer, Tohoku University (Sendai-shi, Miyagi, Japan), as described previously [14]. The cultures were maintained in RPMI-1640 medium and supplemented with 10% fetal bovine serum for no longer than 12 weeks after recovery from frozen stocks. Culture conditions were 37 °C under humidified 5% CO_2_. 

### 4.4. Tumor Growth

**Figure 5:**
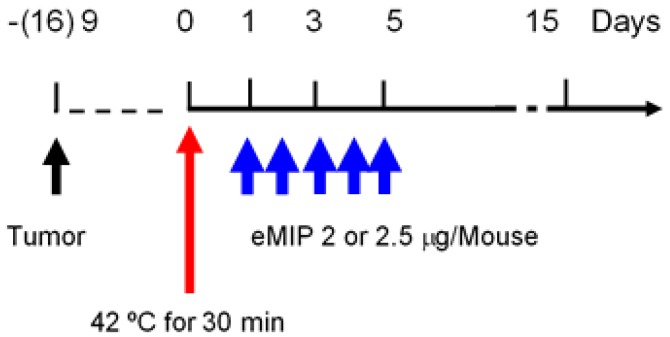
Experimental schedule.

Before implantation, cells were trypsinized and filtered through a cell strainer (70-μm pores). Colon26 adenocarcinoma cells were implanted subcutaneously in the right hind leg (1 × 10^5^ cells/mouse, *n* = 6 per group) or subcutaneously in the right flank of BALB/c mice (2 × 10^5^ cells/mouse, *n* = 7 per group). After 9 or 16 days when tumor size reached ~11 mm, mice with similar-sized tumors were selected and used for the experiments (Figure 5). Tumor size was measured using calipers and tumor volume was calculated as: (major axis) × (minor axis)^2^ × 0.5236.

### 4.5. Pulmonary Metastasis Assay

Four weeks after tumor cell implantation in the right hind leg, bilateral lungs of the mice were fixed overnight with Bouin’s solution. The metastatic nodules on the surfaces of all the pulmonary lobes were counted macroscopically.

### 4.6. HT and eMIP Administration

When sizes of solid tumors in mice reached ~11 mm in diameter, they were exposed to electromagnetic waves to elevate and maintain intratumoral temperature or skin surface temperature at 42 °C for 30 min. Either an 8-MHz capacitive radio-frequency device (Thermotron-RF8, Yamamoto Vinita Co., Ltd., Osaka-shi, Osaka, Japan) or an in-house heating device that uses electromagnetic waves were employed. In the latter case, skin surface temperature was monitored by using a disposable body surface temperature probe (Philips: Tokyo Japan). HT treatment was not repeated, since we focused on the effect of eMIP after heat treatment. eMIP (2 or 2.5 µg/mouse/day) was administered intravenously for 5 consecutive days starting from the next day after HT treatment (Figure 5). 

### 4.7. Statistical Analysis

Significance of differences was tested by one-way ANOVA, followed by Dunnett’s multiple comparison test, using GraphPad Prism version 8.1.1 for Mac OS X (GraphPad Software, San Diego, CA, USA). A probability value of *p* < 0.05 for mean values was considered significant.

## 5. Conclusions

In this study, we present data showing that a chemokine CCL3 derivative, eMIP, administered after HT treatment produces marked tumor growth inhibition and dramatic reduction of distant metastases. It is likely that clinical application of these findings has implications for cancer treatment, increasing the survival rate of patients with metastatic cancers. 

## References

[1] Falk M.H., Issels R.D. (2001). Hyperthermia in oncology. Int. J. Hyperth..

[2] Oldenborg S., van Os R., Oei B., Poortmans P. (2019). Impact of technique and schedule of reirradiation plus hyperthermia on outcome after surgery for patients with recurrent breast cancer. Cancers (Basel).

[3] Helderman R., Loke D.R., Kok H.P., Oei A.L., Tanis P.J., Franken N., Crezee J. (2019). Variation in clinical application of hyperthermic intraperitoneal chemotherapy: A review. Cancers (Basel).

[4] Kaur P., Hurwitz M.D., Krishnan S., Asea A. (2011). Combined hyperthermia and radiotherapy for the treatment of cancer. Cancers (Basel).

[5] Sato T., Sawaji Y., Matsui N., Sato H., Seiki M., Mori Y., Ito A. (1999). Heat shock suppresses membrane type 1-matrix metalloproteinase production and progelatinase A activation in human fibrosarcoma HT-1080 cells and thereby inhibits cellular invasion. Biochem. Biophys. Res. Commun..

[6] Hanahan D., Weinberg R.A. (2011). Hallmarks of cancer: The next generation. Cell.

[7] Mahmood J., Shukla H.D., Soman S., Samanta S., Singh P., Kamlapurkar S., Saeed A., Amin N.P., Vujaskovic Z. (2018). Immunotherapy, radiotherapy, and hyperthermia: A combined therapeutic approach in pancreatic cancer treatment. Cancers (Basel).

[8] Hiniker S.M., Maecker H.T., Knox S.J. (2015). Predictors of clinical response to immunotherapy with or without radiotherapy. J. Radiat. Oncol..

[9] van der Burg S.H., Arens R., Ossendorp F., van Hall T., Melief C.J. (2016). Vaccines for established cancer: Overcoming the challenges posed by immune evasion. Nat. Rev. Cancer.

[10] Feng H., Zeng Y., Graner M.W., Katsanis E. (2002). Stressed apoptotic tumor cells stimulate dendritic cells and induce specific cytotoxic T cells. Blood.

[11] Frey B., Weiss E.M., Rubner Y., Wunderlich R., Ott O.J., Sauer R., Fietkau R., Gaipl U.S. (2012). Old and new facts about hyperthermia-induced modulations of the immune system. Int. J. Hyperth..

[12] Tsang Y.W., Huang C.C., Yang K.L., Chi M.S., Chiang H.C., Wang Y.S., Andocs G., Szasz A., Li W.T., Chi K.H. (2015). Improving immunological tumor microenvironment using electro-hyperthermia followed by dendritic cell immunotherapy. BMC Cancer.

[13] Shiraishi K., Ishiwata Y., Nakagawa K., Yokochi S., Taruki C., Akuta T., Ohtomo K., Matsushima K., Tamatani T., Kanegasaki S. (2008). Enhancement of antitumor radiation efficacy and consistent induction of the abscopal effect in mice by ECI301, an active variant of macrophage inflammatory protein-1alpha. Clin. Cancer Res..

[14] Kanegasaki S., Matsushima K., Shiraishi K., Nakagawa K., Tsuchiya T. (2014). Macrophage inflammatory protein derivative ECI301 enhances the alarmin-associated abscopal benefits of tumor radiotherapy. Cancer Res..

[15] Adkins I., Sadilkova L., Hradilova N., Tomala J., Kovar M., Spisek R. (2017). Severe, but not mild heat-shock treatment induces immunogenic cell death in cancer cells. Oncoimmunology.

[16] Sporn M.B. (1996). The war on cancer. Lancet.

[17] Shoji H., Motegi M., Takakusagi Y., Asao T., Kuwano H., Takahashi T., Ogoshi K. (2017). Chemoradiotherapy and concurrent radiofrequency thermal therapy to treat primary rectal cancer and prediction of treatment responses. Oncol. Rep..

[18] Das P., Colombo M., Prosperi D. (2019). Recent advances in magnetic fluid hyperthermia for cancer therapy. Colloids Surf. B Biointerfaces.

[19] Suzuki K. (1967). Application of heat to cancer chemotherapy--experimental studies. Nagoya J. Med. Sci..

[20] Dickson J.A., Suzangar M. (1974). *In vitro*-*in vivo* studies on the susceptibility of the solid Yoshida sarcoma to drugs and hyperthermia (42 degrees). Cancer Res..

[21] Barlogie B., Corry P.M., Drewinko B. (1980). In vitro thermochemotherapy of human colon cancer cells with cis-dichlorodiammineplatinum(II) and mitomycin C. Cancer Res..

[22] Shiu M.H., Cahan A., Fogh J., Fortner J.G. (1983). Sensitivity of xenografts of human pancreatic adenocarcinoma in nude mice to heat and heat combined with chemotherapy. Cancer Res..

[23] Tsumura M., Yoshiga K., Takada K. (1988). Enhancement of antitumor effects of 1-hexylcarbamoyl-5-fluorouracil combined with hyperthermia on Ehrlich ascites tumor in vivo and Nakahara-Fukuoka sarcoma cell *in vitro*. Cancer Res..

[24] Hahn G.M., Hahn G.M. (1982). Responses of murine tumors and normal tissues. Hyperthermia and Cancer.

